# 663. Vitamin D Deficiency as a Risk Factor for Hypertrophic Scarring and Reconstructive Services After Burns

**DOI:** 10.1093/jbcr/irag033.529

**Published:** 2026-04-06

**Authors:** Philong Nguyen, Yousef Tanas, Joshua Wang, Kamryn K Baker, Alexandra M Dunker, Kevin Vargas, Sudhanvan Iyer, Noelle Gonzalez, Cameron Bowers, Jong Lee

**Affiliations:** University of Texas Medical Branch, Galveston, TX; Houston Methodist Hospital, Houston, TX; University of Texas Medical Branch, Galveston, TX; McGovern Medical School at University of Texas Health Science Center at Houston, Houston, TX; McGovern Medical School at University of Texas Health Science Center at Houston, Houston, TX; Baylor College of Medicine, Houston, TX; University of Texas Medical Branch, Galveston, TX; University of Texas Medical Branch - John Sealy School of Medicine, Houston, TX; University of Texas Medical Branch, Galveston, TX; Division of Burn and Trauma Surgery, Department of Surgery, University of Texas Medical Branch, Galveston, Galveston, TX

## Abstract

**Introduction:**

Vitamin D plays an important role in immune regulation, collagen remodeling, and wound healing. In the general population, deficiency has been associated with poor wound repair and fibrosis, yet evidence in burn patients remains limited. Burn injuries often result in hypertrophic scarring and significant healthcare utilization for reconstructive and adjunctive procedures. This study examines the impact of preexisting vitamin D deficiency on hypertrophic scarring and scar-related healthcare utilization in a large, multi-institutional cohort of burn patients.

**Methods:**

The TriNetX Research Network was queried for patients ≥18 years old with burn injuries between 2010 and 2023. Patients with vitamin D deficiency, defined as 25-hydroxyvitamin D < 20 ng/mL within one month prior to injury, were matched 1:1 to non-deficient controls using propensity score matching. Matching variables included demographics, body mass index, comorbidities, substance use, vitamin deficiencies (A, C, E, and K), laboratory calcidiol levels, and total burn surface area. The primary outcome was hypertrophic scarring. Secondary outcomes included scar-related interventions: Z-plasty, intralesional corticosteroid injections, and laser therapy. Cumulative incidence was assessed at 3, 12, and 24 months. Hazard ratios (HRs) with 95% confidence intervals (CIs) were calculated using Cox proportional hazards models.

**Results:**

After matching, 6832 patients were included in each cohort. Vitamin D deficiency was associated with significantly higher rates of hypertrophic scarring at 3 months (5.82% vs 2.55%, HR 2.33, 95% CI 1.93–2.81, *p*<.0001), 12 months (9.38% vs 4.56%, HR 2.12, 95% CI 1.84–2.46, *p*<.0001), and 24 months (10.61% vs 5.26%, HR 2.09, 95% CI 1.83–2.40, *p*<.0001). Deficient patients also demonstrated greater use of reconstructive services, including Z-plasty (HR 1.86–2.38), corticosteroid injection (HR 3.14–3.56), and laser therapy (HR 1.44–1.55).

**Conclusions:**

Vitamin D deficiency is strongly associated with increased hypertrophic scarring and greater reliance on reconstructive and adjunctive procedures following burn injury. These results underscore the potential role of micronutrient status in long-term burn recovery. Prospective studies are needed to determine whether early detection and correction of deficiency can mitigate scarring and reduce healthcare utilization.

**Applicability of Research to Practice:**

Routine screening and treatment of vitamin D deficiency in burn patients may represent a modifiable target to improve scarring outcomes and reduce the burden of secondary interventions.

**Funding for the study:**

This study was supported by university funding through the Institute for Translational Sciences (UL1 TR001439), funded by the National Center for Advancing Translational Sciences at the NIH.

